# Deaths and injuries from constructions: a call for safer buildings

**DOI:** 10.1016/j.lana.2025.101128

**Published:** 2025-05-09

**Authors:** The Lancet Regional Health – Americas

Disasters due to collapse of man-made structures are not an uncommon problem globally. However, they are not considered a major issue in public health. On April 8, 2025, a nightclub roof collapsed and caused at least 221 deaths and left more than 180 people injured in Santo Domingo, Dominican Republic. This tragedy occurred six weeks after another accident happened in Trujillo, Peru, where the collapse of a food court roof killed six people and injured 78 others. In the USA, the most recent and largest structural disaster occurred in June, 2021, in Miami, FL, USA, where a 12-story condominium partially collapsed, killing 98 people and injuring 11 others. In October, 2024, a 10-story hotel in Buenos Aires Province, Argentina, collapsed and killed at least eight people. These recent events are a reminder that people's lives are constantly vulnerable and exposed to structures that could suddenly fail.

Often, and unfortunately, the occurrence of this type of disaster has an anecdotal connotation for national or even local governments, with little or no effect on reforms of current policies that should otherwise be escalated proportionally to the magnitude of the tragedies. While collapses of buildings, bridges, and other public places are more common among structural disasters, resulting in casualties and injuries, dam collapses often result in substantially larger death tolls. For example, the 1889 collapse due to heavy rain of the South Fork dam in PA, USA, killed more than 2200 people. Dam-related disasters are one of the deadliest civil-engineering catastrophes in the world. In 1975, the collapse of a series of dams in Henan, China killed between 25 000 and 240 000 people, causing the major flooding disaster ever documented.

In addition to deaths and injuries, structural collapse could also have other effects on health, from respiratory diseases due to air pollution to mental health conditions including post-traumatic stress disorder. These conditions can persist for years, as in the case of the long-term effects observed in individuals affected by the attack on the World Trade Center. Water and food insecurity could also be a problem in the case of massive disasters such as dam failures. Similarly to natural disasters, an interdisciplinary response is required, and health-care system preparedness is key to assist injured people. Likewise, effective policies and contingency plans to respond to emergencies require research data, an aspect that is needed in this field, just as the scientific community needs data to better understand the impact of natural disasters and climate change on health and mortality.

There are commendable examples of government response to structural disasters. For instance, 11 months after the condominium collapse occurred in Miami, the SB 4-D — Building Safety bill became law in the state of Florida so all tall buildings must now have milestone inspections. However, it would be even better to implement strict preventive measures for safer buildings before a disaster occurs, to minimize the occurrence of future disasters and avert casualties, injuries, and related health conditions. We also need safer buildings for construction workers. Civil engineers, in collaboration with public health specialists, should ensure the highest standards in planning, design, and construction of safe structures. Public health experts in environmental health should carefully monitor potential health risks such as asbestos and lead in construction materials. Saving money by hiring less-skilled personnel, using poor-quality materials, and promoting lax regulations should be forbidden. Although every city has departments responsible for building safety inspections, more needs to be done. More frequent and thorough inspections are needed. Proper routes of escape in public buildings could save lives in emergencies such as fire, or even in the case of structural collapses. In 2013, 242 people were killed and more than 630 others injured in a fire in a nightclub in Rio Grande do Sul, Brazil. The building had only one exit, which probably contributed to the high death toll. Lack of proper escape routes is still a common issue among infrastructures across the world, more so in low-income and middle-income countries.

A great deal of attention in public health and the scientific community has focused on natural disasters. However, disasters related to man-made structures often receive insufficient attention despite reports show the number of accidents and people affected is not trivial. Coordination of public health specialists with policy makers and governments should be strengthened, targeting prevention and enforcing current policies. Inadequate efforts to foster stricter regulations for new constructions and inspections of existing structures will perpetuate the high frequency of structural collapses. Accountability should also be enforced. We need rigorous, up-to-date policies, policy compliance, sufficient qualified workforce, urban planning, and empiric evidence to better prevent structural collapses, even more so in an era of rapid population growth with increased demand for residential lands. We should call for stricter safety policies and policy compliance not only in the Americas but globally.

